# Comparison of molecular profiles (Nectin-4 and TROP-2) in upper tract urothelial carcinoma with a positive history of urinary bladder cancer vs. UTUC only in the era of ADCs

**DOI:** 10.1186/s12885-025-15042-7

**Published:** 2025-10-06

**Authors:** Mohammed Rafea Kanaan, Jessica Schmitz, Jan Hinrich Braesen, Markus Antonius Kuczyk, Hossein Tezval

**Affiliations:** 1 Department of Urology and Urological Oncology, Hanover Medical School (MHH), Hanover, 30625 Germany; 2 Department of Pathology, Hanover Medical School (MHH), Hanover, 30625 Germany

**Keywords:** TROP-2 (trophoblast cell surface antigen 2), Nectin-4, Upper tract urothelial carcinoma (UTUC), Urinary bladder cancer (UBC), Immunohistochemical analysis (IHC)

## Abstract

Upper tract urothelial carcinoma (UTUC) and urinary bladder cancer (UBC), though histologically similar, differ molecularly, prompting interest in their biomarker profiles for targeted therapies like antibody-drug conjugates (ADCs) enfortumab vedotin (targeting Nectin-4) and sacituzumab govitecan (targeting TROP-2). This study investigated Nectin-4 and TROP-2 expression in 87 UTUC patients, including 54 with a history of concurrent UBC (UTUC + UBC) and 33 with UTUC alone. Immunohistochemical analysis revealed widespread TROP-2 expression (98.8% of samples), with high levels linked to low-grade UTUC (*p* = 0.043) and intense staining (mean H-score 227 ± 63) across both cohorts. Nectin-4 was expressed in 70.1% of samples overall but was more frequent in the UTUC + UBC group (88.8% vs. 63.6% in UTUC-only patients), though this difference lacked statistical significance (*p* = 0.340). Notably, Nectin-4 staining intensity was weak in both groups (mean H-score 66 ± 65), suggesting biological distinctions between UTUC with and without UBC. The findings imply that ADCs targeting TROP-2 and Nectin-4 may hold therapeutic promise in UTUC without requiring prior biomarker testing. Additionally, the elevated Nectin-4 expression in UTUC + UBC patients hints at divergent molecular pathways that could influence treatment strategies, warranting further clinical exploration.

## Introduction

Upper tract urothelial carcinoma (UTUC) is a rare and aggressive subset of urothelial carcinoma, accounting for 5–10% of cases and often presenting with advanced or metastatic disease at diagnosis [1, 2]. This contributes to a poorer prognosis compared to urothelial bladder cancer (UBC) [1, 3]. The phenotypic and genetic differences between UTUC and UBC underline the importance of tailored treatment strategies, as UTUC often demonstrates distinct therapeutic responses [4].

Historically, treatment options for metastatic UTUC have been limited, with poor survival rates observed following platinum-based chemotherapy or immune checkpoint inhibitors. However, recent advances have reshaped the therapeutic landscape. Enfortumab vedotin (EV), an antibody-drug conjugate targeting Nectin-4, has demonstrated significant efficacy in advanced UC, including UTUC, particularly when combined with pembrolizumab, as shown in the pivotal EV-302 trial. This combination therapy improves progression-free survival (PFS) and overall survival (OS), establishing itself as a new standard of care for previously untreated, advanced UC cases [5, 6]. Additionally, sacituzumab govitecan, targeting TROP-2, offers another effective option for patients resistant to earlier treatments [7].

Despite these advancements, the expression profiles of biomarkers like TROP-2 and Nectin-4 in UTUC, particularly in patients with a history of UBC, remain underexplored. Investigating these biomarkers can refine patient selection and support more precise therapeutic approaches. This study focuses on bridging this gap by examining the expression of TROP-2 and Nectin-4 in UTUC patients, contributing to the ongoing development of personalized treatment strategies in precision oncology.

## Materials and methods

### Population and tissue samples

Eighty-seven patients with non-metastatic UTUC were included in the immunohistochemical analysis. The patients underwent radical nephroureterectomy or endoscopic therapy via laser for selected cases with UTUC between January 2010 and December 2023 at Hanover Medical School. Two patient cohorts were established in consideration of the history of urothelial bladder cancer (UBC). The initial group comprised 54 patients (62.1%) with a history of UTUC and UBC, while the second group consisted of 33 patients without a history of UBC. 87 UTUC tissue samples were subjected to immunohistochemical (IHC) analysis to assess the expression of TROP-2 and Nectin-4. This study was conducted in line with the ethical standards outlined in the Declaration of Helsinki and the research integrity policies of Hanover Medical School. Written informed consent was obtained from all patients before recruitment. Given the rarity of UTUC—accounting for only 5–10% of all urothelial carcinomas—and the even lower prevalence of coexisting UTUC with prior UBC, our study includes all eligible patients treated at our institution over a 13-year period. Thus, no formal sample size calculation was feasible, and the cohort size reflects the limitations inherent to studying this rare tumor constellation.

### IHC analysis of Trop-2 and Nectin‑4 expression

IHC staining for NECTIN-4 protein was performed on a VENTANA BenchMark ULTRA autostainer (Ventana) following an accredited protocol in a facility certified to DIN EN ISO/IEC 17,020 standards. Staining was evaluated using the H-score system, calculated as the product of intensity (0–3) and the percentage of stained cells (0–100), as described in the initial study on NECTIN-4 [8]. An H-score >15 was considered positive and further classified into two categories: weak (15–149) or strong (150–300), based on staining intensity after powles et al. [9].

### Statistical analysis

In the first step, a descriptive evaluation was performed. The mean value ± standard deviation was selected for the quantitative parameters, and the absolute or percentage frequency was specified for the individual characteristics of the qualitative aspects. The frequencies for the descriptive illustration were determined with the help of bar charts. The Mann‒Whitney U test for continuous variables or the chi-square test for categorical variables were used to compare the groups.

All the statistical calculations and graphical representations were made with the statistical software IBM-SPSS v. 29.0. All P values were obtained from two-sided statistical tests, with a probability of error of *p* < 0.05 considered to indicate statistical significance.

## Results

### Patient characteristics

Between 2010 and 2023, 87 patients were evaluated and included in the final analysis of this study. The clinicopathological characteristics of the patients are summarized in Table 1. The median age at diagnosis was 70 years, with a male-to-female ratio of 4:1.

In most cases (70.1%), the UTUC was localized in the renal pelvis. Tissue samples were predominantly obtained following radical nephroureterectomy, performed in 85.1% of patients. None of the patients received neoadjuvant chemotherapy before tissue collection.

Regarding the pathological stage of UTUC, the majority of cases were classified as pTa (44.8%) and pNx (54.0%). Additionally, low-grade UTUC was identified in 54.0% of patients. A positive history of urothelial carcinoma of the bladder (UB-Ca) was observed in 62.1% of the patients.

**Table 1:** Patient characteristics

Age (years), mean ± SD		70 ± 10	
sex (m/w), n (%)		64 (74%)/23 (26%)	
Type of operation, n (%)	open surgery	46 (52,9%)	
	minimally invasive surgery	28 (32,2%)	
	endoscopic laser therapy	13 (14,9%)	
Localization (pelvis/ureter), n (%)		61 (70,1%)/26 (29,9%)	
UB-Ca, n (%)		54 (62%)	
Side (right/left), n (%)		50 (57,5%)/37 (42,5%)	
Pathology:			
T-Staging	Ta		39 (44,8%)
	T1		16 (18,4%)
	T2		7 (8,0%)
	T3		20 (23,0%)
	T4		3 (3,4%)
	(+) Tis		5 (5,7%)
N-Staging	Nx		62 (71,2%)
	N0		20 (23,0%)
	N1		1 (1,1%)
	N2		4 (4,6%)
Grading	Low grade		47 (54,0%)
	High grade		40 (46,0%)
R-Staging	Rx		13 (14,9%)
	R0		71 (81,6%)
	R1		3 (3,4%)

### Nectin-4 expression and association with clinicopathological characteristics

**Fig. 1:**
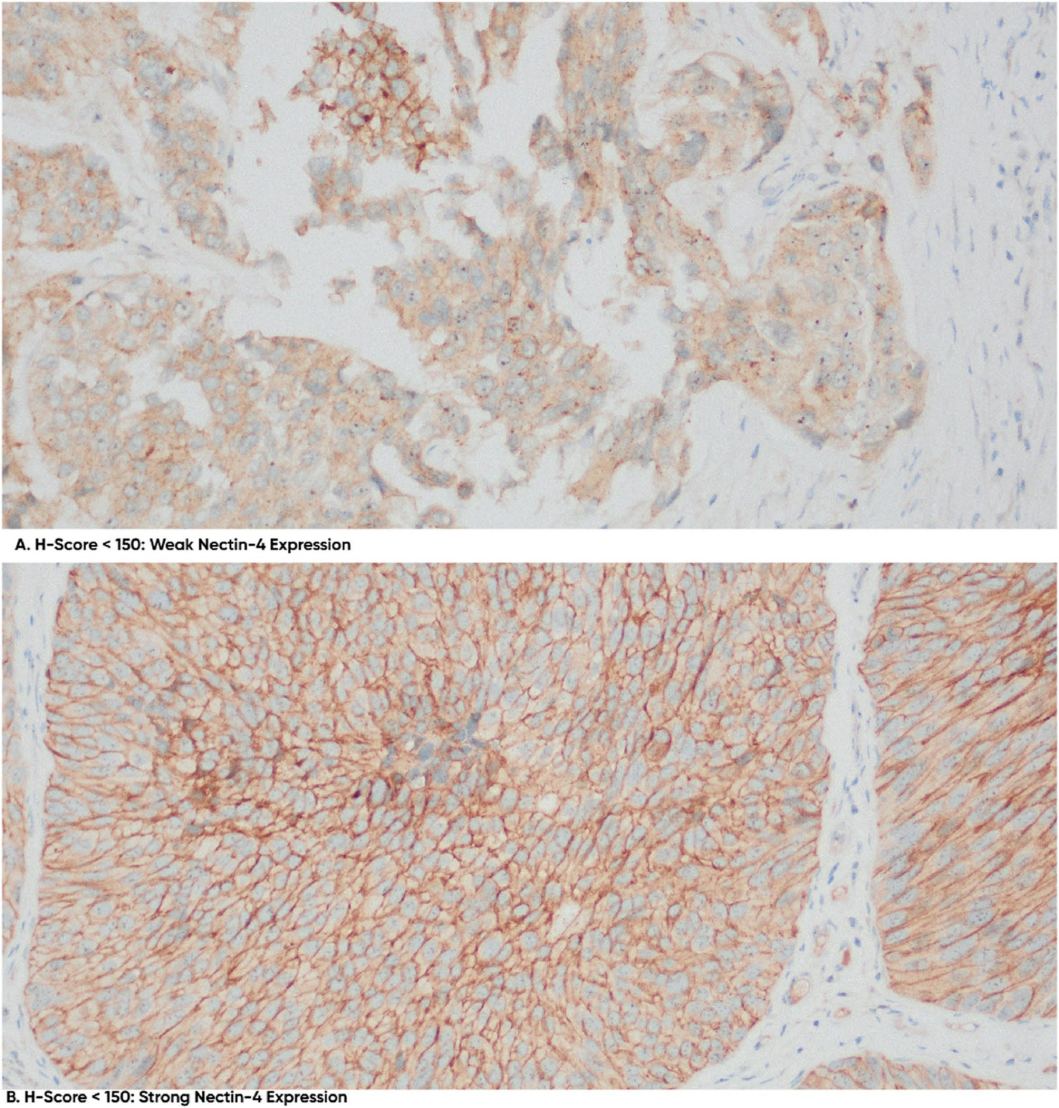
Immunohistochemical analysis of Nectin-4 expression in upper tract urothelial carcinoma (UTUC). Representative IHC-stained sections demonstrating variable Nectin-4 expression in UTUC tissue samples

Positive Nectin-4 expression, as shown in Fig. 1, was observed in 61 out of 87 UTUC samples (70.1%), with a mean H-Score of 66. The UTUC group with a positive history of UB-Ca exhibited a higher expression rate (74.1%) compared to the UTUC-only group (63.6%), though this difference did not reach statistical significance (*p* > 0.05). In binary logistic regression, Nectin-4 expression showed a non-significant association with diagnostic group (OR 1.63, 95% CI 0.64–4.16, *p* = 0.304), indicating a possible but statistically inconclusive trend toward higher expression in UTUC + UBC cases.

The intensity of Nectin-4 expression was low in most cases, with 48 out of 61 positive samples (78.7%) showing low-intensity expression. No significant differences in intensity were found between the diagnostic groups, with 76.2% of UTUC-only cases and 80.0% of UTUC cases with a history of UB-Ca exhibiting low intensity (*p* > 0.05).

Our analysis of the association between Nectin-4 expression (and its intensity) and clinicopathological features across the diagnostic groups (Table 2) revealed no significant correlations with patient sex, age, tumor location, tumor grade, lymphovascular invasion, or lymph node metastasis.

**Table 2:** Immunohistochemical analysis of Nectin-4 and TROP-2 in patients with UTUC

Immunohistochemical Analysis:		
Nectin-4:		
Nectin-4 H-Score (Mean ± SD)		66 ± 65
Nectin-4 Expression (H-Score > 15), n (%)		61 (70,1%)
Nectin-4 Intensity, n (%)	weak (H-Score < 150)	48 (78,7%)
	strong (H-Score ≥ 150)	13 (21,3%)
TROP-2:		
TROP-2 H-Score (Mean ± SD)		227 ± 63
TROP-2 Expression (H-Score > 15), n (%)		86 (98.8%)
TROP-2 Intensity, n (%)	weak (H-Score < 150)	10 (11,5%)
	strong (H-Score ≥ 150)	76 (87,4%)

### Trop-2 expression and association with clinicopathological characteristics

Positive TROP-2 expression was detected in 86 out of 87 UTUC samples (98.8%), with a higher mean H-Score of 227 compared to Nectin-4. No differences in TROP-2 expression were observed between the two diagnostic groups.

**Fig. 2:**
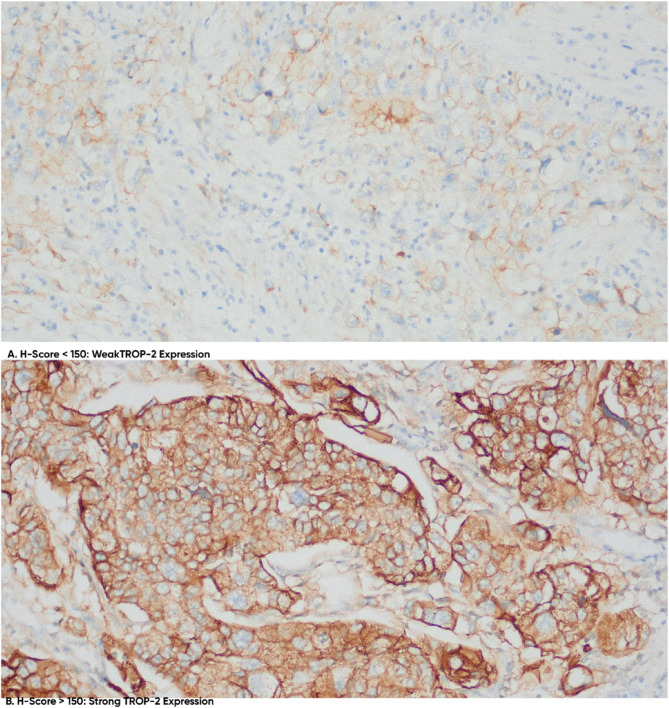
Immunohistochemical analysis of TROP-2 expression in upper tract urothelial carcinoma (UTUC). Representative IHC-stained sections demonstrating variable TROP-2 expression in UTUC tissue samples

The intensity of TROP-2 expression was vigorous in 76 of the 86 cases (87.4%). Among these, the UTUC group with a positive history of UB-Ca exhibited a higher intensity of expression (90.7%) than the UTUC-only group (84.4%). However, this difference did not reach statistical significance (*p* > 0.05). Figure 2 shows the differences in the intensity of TROP-2 expression.

Our analysis of TROP-2 expression and its intensity across the 86 UTUC patients (Table 2) identified a significant association between tumor grade and TROP-2 expression intensity. Specifically, low-grade UTUC displayed stronger TROP-2 expression (95.6%) compared to high-grade UTUC (80.5%), with the difference achieving statistical significance (*p* = 0.04). However, no significant associations were found between TROP-2 expression and other clinicopathological factors, such as patient sex, age, tumor location, lymphovascular invasion, or lymph node metastasis.

### Subgroup analysis of Nectin-4 and TROP-2 intensity after risk classification of U-BC

Based on the European Association of Urology (EAU) classification of non-muscle-invasive bladder cancer (NMIBC), we categorized patients with a positive history of UB-Ca into risk groups. Two distinct groups were established: the first group included patients with low- and intermediate-risk UB-Ca (*n* = 28), while the second group comprised those with high- and very-high-risk UB-Ca (*n* = 26).

Among patients in the higher-risk group of UB-Ca with positive Nectin-4 expression (*n* = 22), the intensity of Nectin-4 expression was more pronounced (27.3%) compared to the low- and intermediate-risk group (11.1%). However, this difference did not reach statistical significance (*p* > 0.05). Conversely, the intensity of TROP-2 expression did not differ significantly between the two subgroups, with expression rates of 89.3% and 92.3%, respectively (see Table 3 below).

**Table 3:** Nectin-4 and TROP-2 intensity after risk classification of U-BC

	Diagnosis Group			
		UTUC	UTUC + low/intermediate risk UB-Ca	UTUC + high risk UB-Ca
Nectin-4 Intensity, n (%)	weak (H-Score < 150)	16 (33%)	16 (33%)	16 (33%)
	strong (H-Score ≥ 150)	5 (38%)	2 (15%)	6 (46%)
TROP-2 Intensity, n (%)	weak (H-Score < 150)	5 (50%)	3 (30%)	2 (20%)
	strong (H-Score ≥ 150)	27 (36%)	25 (33%)	24 (32%)

## Discussion

This study aimed to compare the expression levels of TROP-2 and Nectin-4 in patients with upper tract urothelial carcinoma (UTUC) who had a prior history of urinary bladder carcinoma (UB-Ca) to those without such a history. While TROP-2 and Nectin-4 are well-studied therapeutic targets in UB-Ca, their comparative expression patterns in UTUC subgroups remain underexplored, particularly in patients with a history of UB-Ca. Our findings addressed this gap and highlighted critical differences with potential clinical implications.

We demonstrated that Nectin-4, the target protein of enfortumab vedotin, was expressed in 70.1% of patients with UTUC. This finding is consistent with the results of Tomiyama et al., who reported an expression rate of 65.7% in their cohort [10]. In UB-Ca, however, Nectin-4 expression has been documented in approximately 83% of cases, suggesting a slightly higher expression rate than UTUC [11]. Intriguingly, UTUC patients with prior UB-Ca history showed a non-significantly higher Nectin-4 expression rate (74.1% vs. 63.6%, *p* > 0.05). This trend mirrors findings by Klümper et al., who observed reduced Nectin-4 expression in metastatic urothelial carcinoma, potentially linked to EV resistance mechanisms [8]. While statistical significance was not achieved, this trend underscores the need for larger cohort studies to clarify the role of UB-Ca history in UTUC biology.

Powles et al. previously reported a median H-Score of 280 in patients with locally advanced or metastatic urothelial cancer, without distinguishing between UTUC and UB-Ca, in their exploratory analysis of Nectin-4 expression’s impact on outcomes with enfortumab vedotin (EV) plus pembrolizumab (P) in the Phase 3 EV-302 study [9]. Our cohort, however, showed markedly lower H-scores (mean 66), with 78.7% of patients falling into low-expression categories. This discrepancy may reflect both biological and methodological factors. Biologically, UTUC may inherently exhibit lower Nectin-4 expression due to its distinct urothelial differentiation like tumor stage (our cohort included localized UTUC vs. EV-302’s metastatic cases), immune context, or tumor evolution compared to bladder tumors [12–14]. Methodologically, variations in tissue fixation, antibody clones, or H-score evaluation across studies may contribute to the observed differences [13]. These findings emphasize the importance of tumor-site- and disease-stage-specific validation of Nectin-4 as a therapeutic biomarker before applying ADC-based treatment strategies. Despite the H-score widespread application, establishing standardized H-score thresholds for clinical responses remains a subject of debate, primarily due to variability in scoring systems across different institutions.

Recent studies have shown that TROP-2, the target protein of sacituzumab govitecan, is widely expressed in UTUC, with 94% of UTUC cases demonstrating positivity. High TROP-2 expression has also been associated with favorable prognosis in UTUC [15]. In our study, TROP-2 expression was detected in 98.8% (86 out of 87 patients), confirming the high expression rate of TROP-2 in UTUC, which is higher than that of Nectin-4. Notably, our findings were consistent with Tomiyama et al., who observed stronger TROP-2 expression (95.6%) in low-grade UTUC compared to high-grade variants, which was associated with a favorable prognosis [15]. This contrasts with findings in other cancers, such as non-muscle-invasive UB-Ca, breast cancer, and metastatic prostate cancer, where high TROP-2 expression has been linked to increased tumor aggressiveness and poor prognosis [15–20]. Recent UTUC studies indicate that high TROP-2 expression may instead reflect a more differentiated, luminal-like phenotype associated with favorable outcomes in this tumor type [15]. Differences in subcellular localization (membranous vs. cytoplasmic), signaling partners, and co-expression with luminal markers may modulate its function and prognostic implications in UTUC [15, 20]. In our cohort, no significant associations were found between TROP-2 expression and clinicopathological factors such as lymphovascular invasion or lymph node or distant metastasis.

Additionally, subgroup analysis based on UB-Ca classification did not reveal significant differences in Nectin-4 or TROP-2 expression. Although patients with a positive history of UB-Ca exhibited higher intensity of TROP-2 expression, the difference was not statistically significant. This suggests that a history of UB-Ca does not directly influence the expression levels of TROP-2 or Nectin-4 in UTUC.

The role of prior intravesical Bacillus Calmette–Guérin (BCG) treatment as a potential modifier of phenotypic marker expression, including Nectin‑4 and TROP‑2, remains understudied in UTUC. In non‑muscle‑invasive bladder cancer (NMIBC), multiple transcriptomic analyses have shown that both Nectin‑4 and TROP‑2 expression levels generally remain stable following BCG therapy, suggesting limited direct modulation by this immunotherapy [21]. However, another molecular study reported an upregulation of these markers post‑BCG exposure in a subset of tumors [22]. These conflicting findings may be influenced by tumor subtype, treatment duration, and intratumoral heterogeneity. In our retrospective UTUC cohort, detailed histories of bladder cancer treatment—including BCG or systemic chemotherapy—were available in only a minority of cases, precluding a robust subgroup analysis. We have therefore acknowledged this as an important limitation and potential confounding factor in interpreting biomarker expression in UTUC cases with prior bladder cancer history.

Our study has several limitations. First, its retrospective design limits causal inference, making it difficult to establish definitive relationships between TROP-2 and Nectin-4 expression and clinical outcomes. Second, the small and imbalanced sample size may limit statistical power. However, this reflects the rarity of UTUC itself—which comprises only 5–10% of urothelial tumors—and the even lower incidence of coexisting UTUC and prior UBC, which constrains prospective cohort size. Third, survival data were unavailable, preventing validation of the prognostic role of TROP-2 and Nectin-4. Fourth, potential prior treatments for UB-Ca, such as intravesical BCG or chemotherapy, may have affected expression of these markers, although such treatment histories were not systematically recorded. Future research should incorporate prospective, multi-institutional cohorts with standardized treatment documentation and centralized pathology review to improve generalizability and biomarker validation.

## Conclusion

This study confirms that TROP-2 and Nectin-4 are widely expressed in UTUC, making them potential therapeutic targets. TROP-2 was more strongly expressed in low-grade UTUC, suggesting its role as a favorable prognostic marker, while Nectin-4 showed no significant correlation with tumor characteristics. Although patients with a history of UB-Ca had slightly higher expression levels of both proteins, the difference was not statistically significant. These findings emphasize the therapeutic importance of TROP-2 and Nectin-4 and highlight the need for further research to clarify their prognostic and predictive value in clinical practice.

## Data Availability

The datasets generated and analysed during the current study are available from the corresponding author on reasonable request.
